# Nucleotide excision repair/transcription gene defects in the fetus and impaired TFIIH-mediated function in transcription in placenta leading to preeclampsia

**DOI:** 10.1186/1471-2164-15-373

**Published:** 2014-05-15

**Authors:** Roxana Moslehi, Xavier Ambroggio, Vijayaraj Nagarajan, Anil Kumar, Amiran Dzutsev

**Affiliations:** 1 Department of Epidemiology and Biostatistics, School of Public Health, University at Albany, State University of New York (SUNY), Rensselaer, NY 12144, USA; 2 Cancer Research Center, University at Albany, SUNY, Rensselaer, NY 12144, USA; 3 Rosetta Design Group LLC, Burlington, VT 05401, USA; 4 Bioinformatics and Computational Biosciences Branch (BCBB), Office of Cyber Infrastructure and Computational Biology (OCICB), National Institute of Allergy and Infectious Disease (NIAID), National Institutes of Health (NIH), Bethesda, MD 20814, USA; 5 Leidos Biomedical Research, Inc, Frederick, MD 21701, USA; 6 Cancer and Inflammation Program, National Cancer Institute (NCI), NIH, Frederick, MD 21702, USA

## Abstract

**Background:**

Preeclampsia is a significant cause of maternal and fetal mortality and morbidity worldwide. We previously reported associations between trichothiodystrophy (TTD) nucleotide excision repair (NER) and transcription gene mutations in the fetus and the risk of gestational complications including preeclampsia. TTD NER/transcription genes, *XPD*, *XPB* and *TTD-A*, code for subunits of Transcription Factor (TF)IIH. Interpreting *XPD* mutations in the context of available biochemical data led us to propose adverse effects on CDK-activating kinase (CAK) subunit of TFIIH and TFIIH-mediated functions as a relevant mechanism in preeclampsia. In order to gain deeper insight into the underlying biologic mechanisms involving TFIIH-mediated functions in placenta, we analyzed NER/transcription and global gene expression profiles of normal and preeclamptic placentas and studied gene regulatory networks.

**Results:**

We found high expression of TTD NER/transcription genes in normal human placenta, above the mean of their expression in all organs. *XPD* and *XPB* were consistently expressed from 14 to 40 weeks gestation while expression of *TTD-A* was strongly negatively correlated (r = -0.7, P < 0.0001) with gestational age. Analysis of gene expression patterns of placentas from a case-control study of preeclampsia using Algorithm for Reconstruction of Accurate Cellular Networks (ARACNE) revealed GTF2E1, a component of TFIIE which modulates TFIIH, among major regulators of differentially-expressed genes in preeclampsia. The basal transcription pathway was among the largest dysregulated protein-protein interaction networks in this preeclampsia dataset. Within the basal transcription pathway, significantly down-regulated genes besides *GTF2E1* included those coding for the CAK complex of TFIIH, namely *CDK7*, *CCNH*, and *MNAT1*. Analysis of other relevant gene expression and gene regulatory network data also underscored the involvement of transcription pathways and identified JUNB and JUND (components of transcription factor AP-1) as transcription regulators of the network involving the TTD genes, *GTF2E1*, and selected gene regulators implicated in preeclampsia.

**Conclusions:**

Our results indicate that TTD NER/transcription genes are expressed in placenta during gestational periods critical to preeclampsia development. Our overall findings suggest that impairment of TFIIH-mediated function in transcription in placenta is a likely mechanism leading to preeclampsia and provide etiologic clues which may be translated into therapeutic and preventive measures.

## Background

Preeclampsia is a complex disorder affecting about 7% of all pregnancies and a major cause of severe intrauterine growth restriction (IUGR) and preterm birth [1]. Preeclampsia has the potential to develop into severe preeclampsia and hemolysis, elevated liver enzymes and low platelets (HELLP) syndrome posing risks of fatality for both mother and infant [2]. While the exact biologic mechanisms leading to preeclampsia remain elusive, abnormal placentation associated with shallow trophoblast invasion and inadequate remodeling of maternal uterine spiral arteries [3,4] followed by placental hypoxia [5] and oxidative stress [6,7], caused by compromised blood flow into the placenta, have been implicated in the underlying pathology of this condition. The intricacies of the involvement of transcription and repair mechanisms in this process have not been elucidated.

We first conceived the hypothesis for the association between nucleotide excision repair (NER) and transcription gene abnormalities in the fetal genome and risk of gestational complications in 2003 based on our clinical observations and systematic genetic epidemiologic investigations in families with trichothiodystrophy (TTD) [8]. TTD is a rare (affected frequency of 1 in 10^6^) recessive disorder [9] caused by mutations in *XPD* (*ERCC2*), *XPB* (*ERCC3*), and *TTD-A* (*GTF2H5*), which encode three of the subunits of transcription factor (TF)IIH, as well as in *TTDN1 *[9-11]. Mutations in *XPB* and *XPD* can cause other rare recessive DNA repair disorders such as xeroderma pigmentosum (XP) [9,12].

TFIIH is a multifunction general transcription factor with roles in both basal and activated transcription as well as in DNA repair. TFIIH is a complex consisting of ten proteins and two domains, a CDK-activating kinase (CAK) domain and a core domain [13]. TFIIH is a component of the RNA Polymerase (Pol)II-mediated transcription machinery [14,15]. Both domains of TFIIH and its interaction with TFIIE are needed for its function in transcription initiation and RNA Pol-II elongation [16]. For its function in the nucleotide excision repair pathway, TFIIH’s core domain alone and its helicase activity via XPD and XPB are needed [17].

Our recent study of XP and TTD suggested a link between TTD- but not XP-associated *XPD* mutations in the fetus and risk of placental maldevelopment and preeclampsia [18]. This study [18] and our subsequent integrative transcriptome analysis [19] implicated impairment of TFIIH-mediated functions in placenta as a possible mechanism involved in preeclampsia. Our meta-analysis of preeclampsia case-control studies suggested that a large number of preeclampsia-specific genes were directly induced by hypoxia and highlighted the signature of oxidative stress in preeclamptic placentas [19]. Given that NER is involved in oxidative damage repair [20], a critical question to resolve would be whether impairment of the NER function of TFIIH could also be relevant to the mechanism leading to preeclampsia.

Our current study focuses on deciphering whether TFIIH’s transcription function alone or a combination of its transcription and repair functions in placenta is relevant to the development of preeclampsia. In order to resolve this question and gain deeper insight into the biologic mechanisms which underlie the association between NER/transcription gene abnormalities in the fetus, impairment of TFIIH-mediated functions in placenta, and preeclampsia, we conducted analyses of several relevant gene expression and gene regulatory network datasets.

## Results

### Analysis of NER/transcription gene profiles in normal human placenta

We analyzed the relative expression of TTD NER/transcription genes in placenta compared to other normal human tissue including skin in GSE96 [21], given that skin abnormalities are among the most prominent features of TTD suggesting high expression of TTD NER/transcription genes in skin. Statistically-significant higher expression was observed for *TTD-A* [False Discovery Rate (FDR) = 0.03, Fold Change = 3.5)] and *XPB* (FDR = 0.06, Fold Change = 5.9) in placenta compared to skin and other tissues. *XPD* expression was also higher in placenta than skin and most other tissues but the difference did not reach statistical significance (Additional file 1: Figure S1a).

Preeclampsia is identified clinically as hypertension that first occurs after 20 weeks gestation [1] but may be due to processes which develop earlier, therefore, genes relevant to preeclampsia would be expected to be expressed during the critical gestational periods of ≤20 weeks gestation as well. In order to infer the relevance of the TTD NER/transcription genes to processes which lead to preeclampsia, we conducted time-course analysis of the three TTD NER/transcription gene expression patterns in normal placenta from 14-40 weeks gestation in GSE5999 [22]. We found high expression of all TTD NER/transcription genes in human placenta from first to third trimester. While *XPD* and *XPB* were consistently expressed from 14 to 40 weeks gestation, expression of *TTD-A* was strongly negatively correlated (r = -0.70, P < 0.0001) with gestational age (Additional file 1: Figure S1b). These results indicate that TTD NER/transcription genes are expressed in placenta at early gestation and during gestational periods which are critical for preeclampsia development.

### Placental gene expression profiles in a preeclampsia case-control study

In order to reconstruct gene regulatory networks of preeclamptic placentas, we analyzed global gene expression profiles of placenta from a large preeclampsia case-control study dataset (GSE 10588) [23]. In our previous meta-analysis of preeclampsia case-control studies [19], we had used gene annotation pathway-based approaches such as gene ontology (GO) [24] to infer regulatory relationships. In our current study, however, we used Algorithm for Reconstruction of Accurate Cellular Networks (ARACNE) [25], a robust information-theory based method specifically developed to reconstruct mammalian cellular gene regulatory networks using gene expression profiles. Annotation-based methods such as GO are limited to previously known relationships, whereas computational-based methods such as ARACNE are more suited for identification of new gene associations and network reconstruction. ARACNE has been shown to outperform several correlation and probabilistic (Bayesian)-based network reconstruction methods [26]. To our knowledge, ARACNE has never been applied to reconstruction of gene networks of preeclamptic placentas.

**Figure 1:**
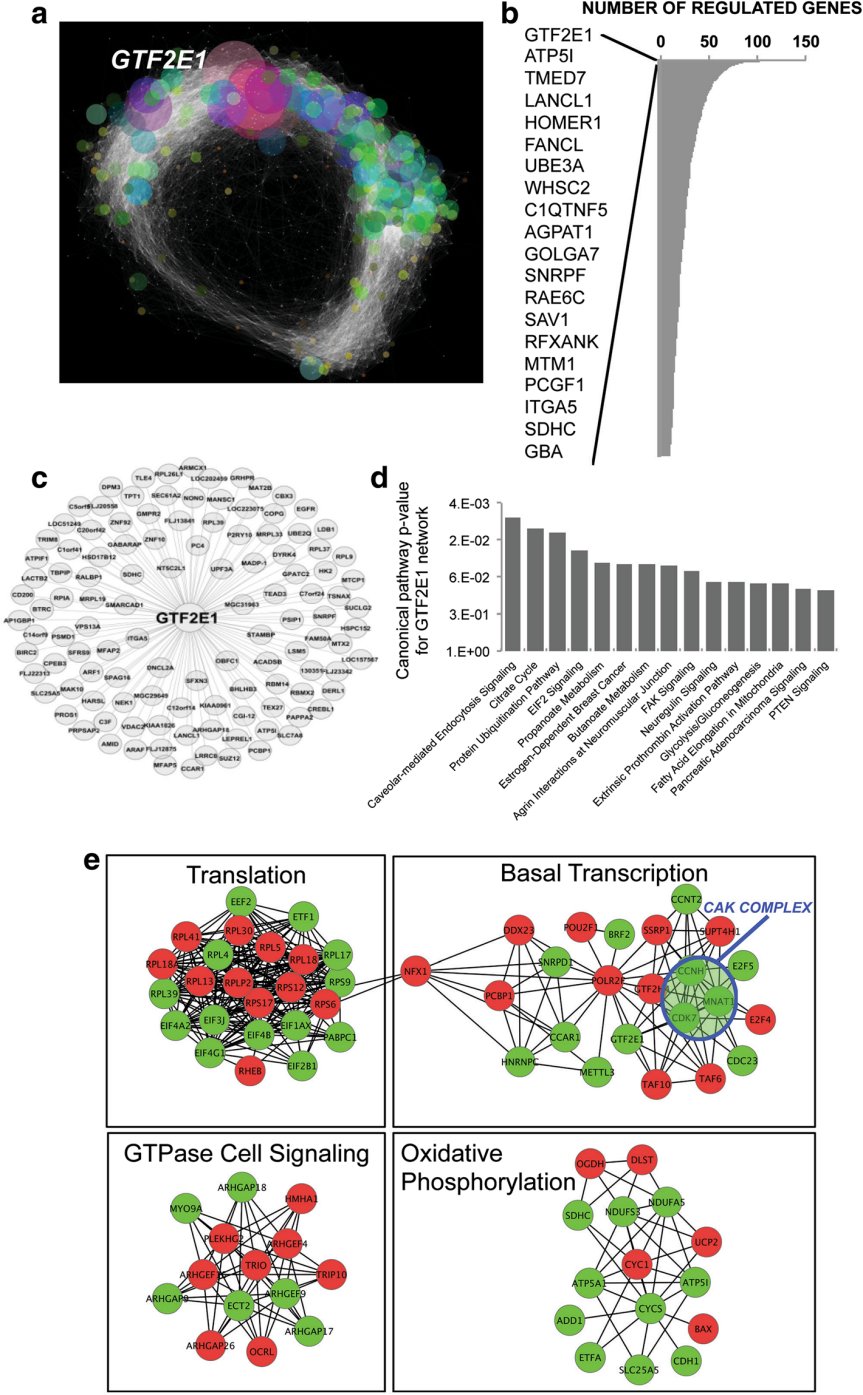
**Placental gene expression profiles in a preeclampsia case-control study (GSE10588) **[23]. Panel **a**. Network of significant connections (edges) between the genes (nodes). Size of each node (representing an expressed gene) is proportional to the number of interactions with other genes and is, therefore, directly proportional to the number of significant associations found. Panel **b**. Bar graph of the number of connections for the most interconnected genes in the network predicted by ARACNE. *GTF2E1* was identified as the gene with the largest number of interactions/connections in the network. Panel **c**. Subnetwork of GFT2E1-regulated genes. Panel **d**. Canonical pathway analysis of GTF2E1-regulated genes. Panel **e**: Cytoscape visualization of dysregulated protein-protein interaction networks. Green nodes signify downregulation and red nodes upregulation of each gene product.

Application of ARACNE to global gene expression profiles of placenta from the largest available preeclampsia case-control dataset fitting our selection criteria (GSE 10588) [23] revealed 2000 genes (i.e., nodes) found to be significant based on their interactions with other genes in the network and 22000 significant interactions (i.e., edges) between the identified genes (Figure 1a). The interconnectivity analysis depicted in Figure 1a points at the complexity of the altered gene regulatory networks of preeclamptic placentas and at *GTF2E1,* which codes for a component of TFIIE, as the most interconnected gene in the network (Figure 1a).

In ARACNE analysis, the most interconnected gene(s) are inferred to be the master regulator(s) in the network defined as gene(s) with the highest number of significant interactions with other genes within the network. In our analysis, the top gene in the network in terms of the largest number of interactions was *GTF2E1* with 120 significant interactions (Figure 1b). Pathway analysis of GTF2E1-regulated genes (Figure 1c) revealed the most prominent canonical pathways as those related to transcription and translation such as nutrient uptake, energy metabolism and protein degradation pathways (Figure 1d). The basal transcription pathway was among the largest dysregulated protein-protein interaction networks in this preeclampsia dataset (Figure 1e). Within the basal transcription pathway, the significantly downregulated genes included *GTF2E1* (FDR = 8.72*10^-7^, Fold Change = 1.7) and those coding for all three components of the CAK complex of TFIIH, namely *CCNH* (FDR = 1.14*10^-5^, Fold Change = 1.7), *CDK7* (FDR = 0.0088, Fold Change = 1.3) and *MNAT1* (which codes for MAT1) (FDR = 7.92*10^-5^, Fold Change = 1.3) (Figure 1e). CAK complex of TFIIH is needed for its transcription function, therefore, downregulation of GTF2E1 and all three subunits of the CAK domain of TFIIH in preeclamptic placentas points towards impairment of TFIIH’s role in transcription.

### Transcriptome analysis of human umbilical vein endothelial cells

**Figure 2:**
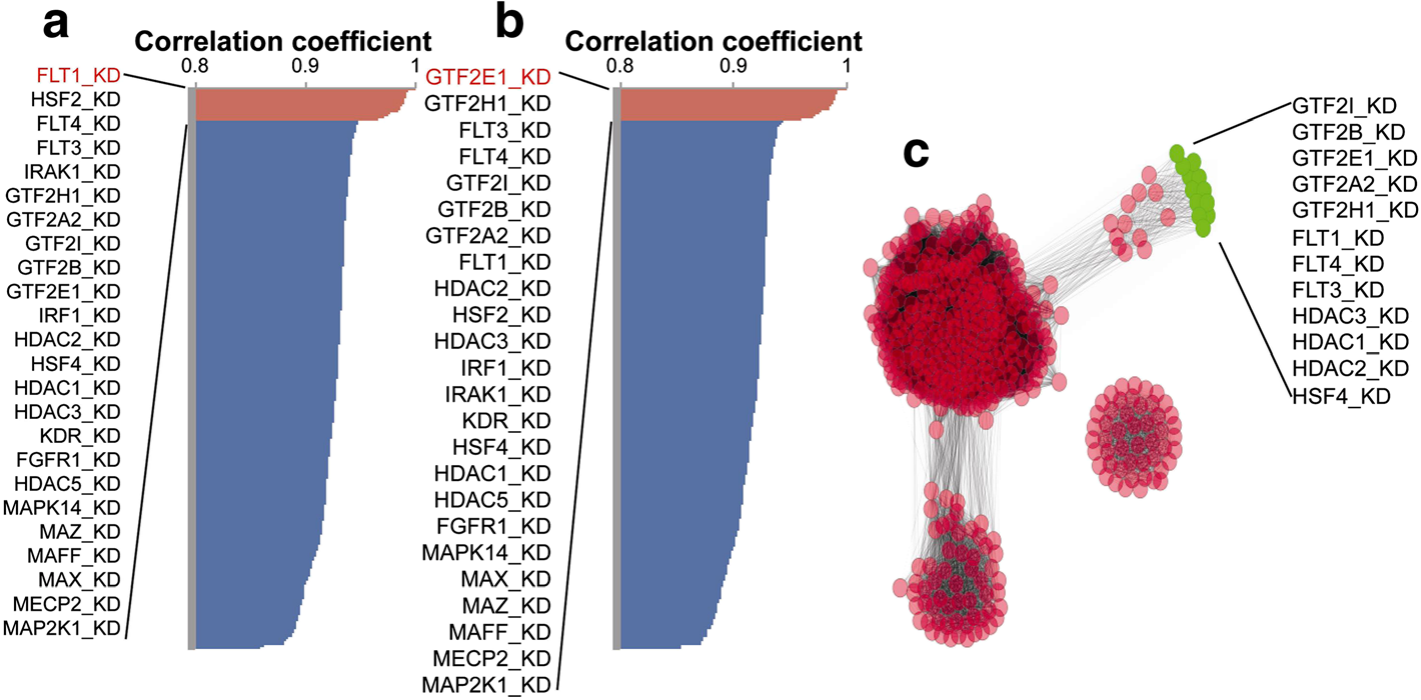
**Analysis of a human umbilical vein endothelial cells dataset (GSE 27871) **[27] **created using siRNA-mediated knockdown of 400 specific signaling molecules and transcription factors.** Panel **a**. Pearson correlation analysis of FLT1 siRNA-mediated knockdown with other datasets revealing a common gene regulatory network of GTF2E1 and FLT1. Panel **b**. Pearson correlation analysis of GTF2E1 siRNA-mediated knockdown with other datasets revealing a common gene regulatory network of GTF2E1 and FLT1. Panel **c**. Correlation matrix of all siRNA-mediated knockdowns revealing significant (r > 0.95) gene clusters. The gene cluster connecting GTF2E1 and FLT1 (i.e., GTF-FLT-HDA-HSF gene cluster) is shown in green.

In order to validate the finding of GTF2E1 as the regulator of the gene network altered in preeclampsia, we analyzed the Human Umbilical Vein Endothelial Cells (HUVEC) transcriptome dataset (GSE27871) [27] to compare the affected gene regulatory networks of GTF2E1 and FLT1 siRNA-inhibited umbilical cord endothelial cells. FLT1 had been identified as a molecular mediator of clinical symptoms of preeclampsia in previous studies [5]. Our analysis of HUVEC dataset identified similarity (p < 10^-6^) between GTF2E1 and FLT1 siRNA datasets suggesting similar consequences for inhibition of FLT1 tyrosine protein kinase and inhibition of transcription in the endothelial cells (Figure 2a and b). Network reconstruction using correlation coefficients (FDR < 0.01) revealed clustering of FLTs with proteins involved in RNA Pol-II holoenzyme, such as general transcription factors (GTFs) and histone deacetylases (HDAC), underscoring the relevance of transcription network (Figure 2c).

### Analysis of relevant datasets to identify common regulatory networks of genes implicated in preeclampsia

**Figure 3:**
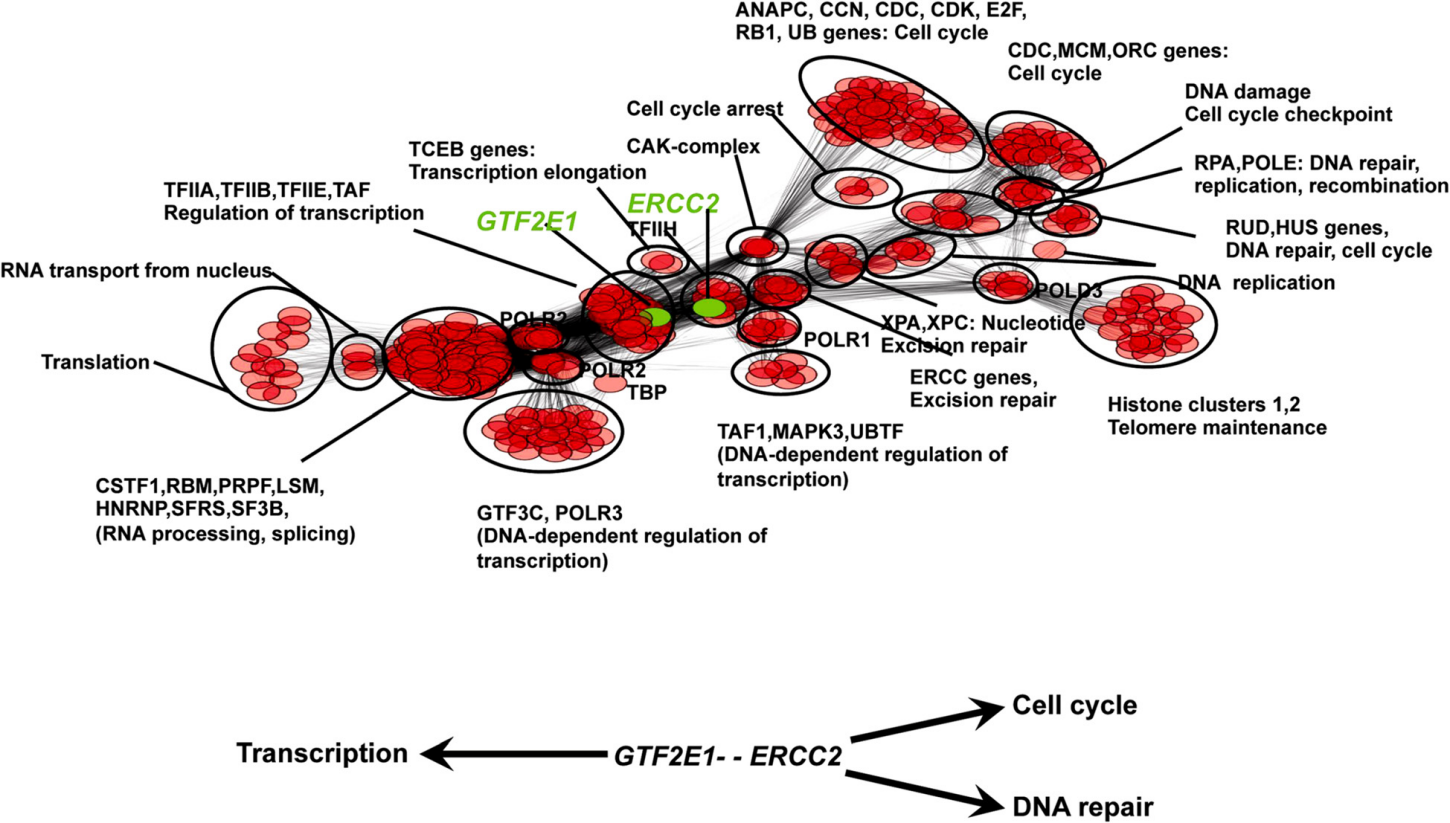
**Network of GTF2E1 interacting proteins.** Network of interacting proteins with up to second-degree connection to GTF2E1 based on data from the reactome dataset [28]. Of note, GTF2E1 has a direct interaction with ERCC2 (XPD). Below the Figure is a graphical summary interpretation depicting the three cellular pathways representing the majority of protein networks with first or second-degree connection to ERCC2 (i.e., Transcription, Cell Cycle, DNA Repair). The GTF2E1-ERCC2 connection highlights the transcription process (depicted in the left side of the figure).

We analyzed the reactome database [28] to identify proteins with direct and indirect (up to secondary) interactions with GTF2E1 and XPD in order to gain clues as to the cellular processes marking their involvement in preeclampsia. This analysis revealed direct interaction between GTF2E1 and ERCC2 (XPD), both of which belong to a highly interactive protein cluster network (Figure 3). In the reactome database, XPD was found to have direct and indirect interactions with proteins involved in several cellular processes including transcription, DNA repair and cell cycle (Figure 3). GTF2E1 was found to interact with proteins involved in transcription. The ERCC2-GTF2E1 connection and its extended (up to secondary interaction) protein interaction network highlighted XPD’s involvement in transcription (depicted in the left side of Figure 3) versus its role in other cellular functions such as repair and cell cycle (depicted in the right side of Figure 3), with respect to the mechanism leading to preeclampsia (Figure 3).

**Figure 4:**
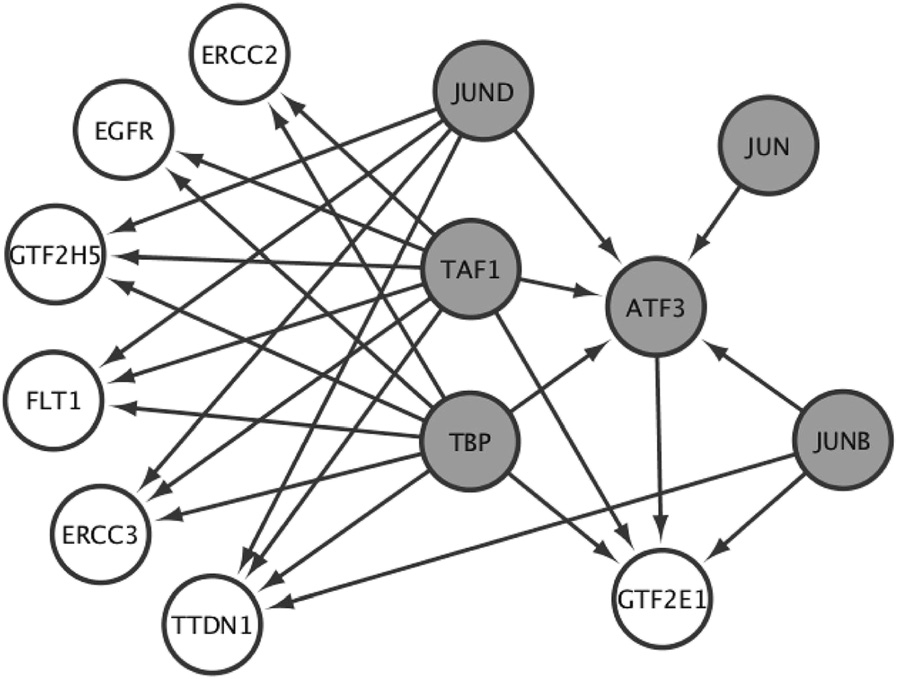
**Analysis of raw ENCODE Chip Seq regulatory network data.** Transcription factors connecting selected genes (such as TTD genes and *GTF2E1*) and gene regulators (such as *EGFR*, *ATF3,* and *FLT1*) implicated in preeclampsia. Grey circles depict genes with Chip Seq information in the ENCODE dataset. Open circles depict genes of interest. JUNB and JUND identified as transcription regulators of all selected factors within the network.

We also conducted an analysis of ENCODE Chip Seq data [29] to infer common transcription regulatory networks of selected genes (such as TTD genes and *GTF2E1*) and gene regulators (such as EGFR [19], ATF3 [19], and FLT1 [5]) implicated in preeclampsia. This analysis revealed a common transcription regulatory network involving *EGFR*, *ATF3*, *FLT1*, *GTF2E1* and TTD genes (i.e., *XPD*, *XPB*, *TTD-A* and *TTDN1*) (Figure 4). These analyses revealed regulation of GTF2E1 by ATF3 and common regulation of XPD and GTF2E1 by TBP (TATA box binding protein), a crucial transcription initiation factor and a subunit of TFIID. JUNB and JUND, components of the AP-1 transcription factor involved in cellular proliferation, migration, differentiation and apoptosis [30], were identified as regulators connecting all genes of interest implicated in preeclampsia (Figure 4), highlighting the relevance of transcription pathways to preeclampsia.

## Discussion

Although singular observations of certain pregnancy complications were noted in individual case reports of TTD patients (as illustrated in our recent genetic epidemiologic study [18]), gestational complications were not recognized as part of the spectrum of TTD multisystem disorder until the initiation of our systematic investigations of the adverse effects of NER/transcription gene abnormalities on fetal development in 2003, as described in the text and authors’ contributions of our previous publication [8]. Our findings have consistently highlighted the relevance of the fetal genotype and the exact genetic abnormality to the mechanism leading to gestational complications such as preeclampsia [8,18].

Based on eight TTD [8,31,32]^a^ and three XP [33-35]^a^ cases with reported *XPD* mutations and explicit information on presence or absence of preeclampsia (many case reports and review articles on XP and TTD lacked explicit information on presence or absence of preeclampsia), we localized the preeclampsia-associated mutations to a C-terminal motif and the helicase surfaces of XPD. Interpreting the *XPD* mutations in the context of available biochemical and epidemiologic data led us to propose that specific subset of *XPD* mutations in the fetus, associated with TTD but not XP, may result in higher risk of preeclampsia due to their adverse effects on binding of XPD with the CAK and p44 subunits of TFIIH, leading to impairment of TFIIH-mediated functions in placenta [18]. As noted in our report [18], a plausible hypothesis based on data analyzed in our study is that impairment of transcription-related functions of TFIIH may be relevant to preeclampsia. Our subsequent integrative transcriptome analysis shed further light on the link between impairment of TFIIH-mediated functions in placenta and induction of molecular mediators of clinical symptoms of preeclampsia through dysregulation of EGFR- and ATF3-dependent pathways [19].

Our current analyses of gene expression patterns from several data sources relevant to placenta and preeclampsia provided additional mechanistic information. We found high expression of TTD NER/transcription genes in normal placenta, above the mean of their expression in all organs including skin where these genes are known to play an important function. Temporal analysis revealed expression of *XPD*, *XPB* and *TTD-A* in placenta throughout second and third trimesters and during critical gestational periods for preeclampsia development. Interestingly, while *XPD* and *XPB* were consistently expressed in placenta from 14 to 40 weeks gestation, expression of *TTD-A* was negatively correlated with gestational age. Preeclampsia was not noted among mothers of patients with mutations in *TTD-A* in our initial genetic epidemiologic study [8,36]. It is unclear how this observation relates to the unique pattern of expression of *TTD-A* in placenta, however, it is plausible that the temporal expression patterns of these genes could provide clues into the genotype-phenotype correlations with respect to gestational complications.

Application of ARACNE to the placental gene expression profiles of the largest available case-control study of preeclampsia fitting our selection criteria (i.e., tissue biopsy from fetal cell-derived histologic subsection of placenta) revealed GTF2E1 (component of TFIIE which modulates TFIIH) among major regulators of differentially-expressed genes in preeclampsia. This finding implicated impaired TFIIE-TFIIH interaction as a relevant mechanism leading to preeclampsia. TFIIH and TFIIE are necessary for accurate transcription; these molecules are believed to mediate the unwinding of DNA as well as melting of DNA to allow RNA Pol-II movement [37]. Our findings in HUVEC suggested the same downstream effects on the gene signature for *GTF2E1* inhibition as that of *FLT1* (i.e., a molecular mediator of preeclampsia symptoms [5]) inhibition with the altered gene signature mimicking preeclampsia development.

Our analysis of placental gene expression patterns in the preeclampsia case-control study also revealed dysregulation of the basal transcription pathway and identified genes coding for the CAK-complex of TFIIH, namely *CDK7*, *CCNH*, and *MNAT1*, among the largest group of downregulated genes in preeclamptic placentas. CDK7 of the CAK complex of TFIIH is required for phosphorylation of RNA Pol-II [14]. XPD is believed to be required for stability of the TFIIH and for coordinating the activity of the CAK complex [12]. Our analyses of the reactome dataset identified direct interaction between GTF2E1 and XPD along with other proteins involved in transcription further pointing towards transcription mechanisms as relevant to preeclampsia. Analysis of ENCODE Chip Seq data confirmed the above findings and suggested that TTD genes, GTF2E1, FLT1 and selected gene regulators of preeclampsia (such as EGFR and ATF3 [19]) may be part of the same protein-protein interaction network. JUNB and JUND, components of transcription factor AP-1 and proto-oncogenes which interact with specific target DNA sequences to regulate gene expression, were identified as regulators of all genes of interest in our network. AP-1 has been implicated in regulation and proliferation of trophoblasts and has been suggested to play a role in the pathogenesis of gestational trophoblastic diseases, which are placental pathologies [38]. *TTDN1,* which has been associated with TTD but does not code for a subunit of TFIIH, was also found to belong to the same gene network regulated by JUNB and JUND, providing possible insight into its function.

**Figure 5:**
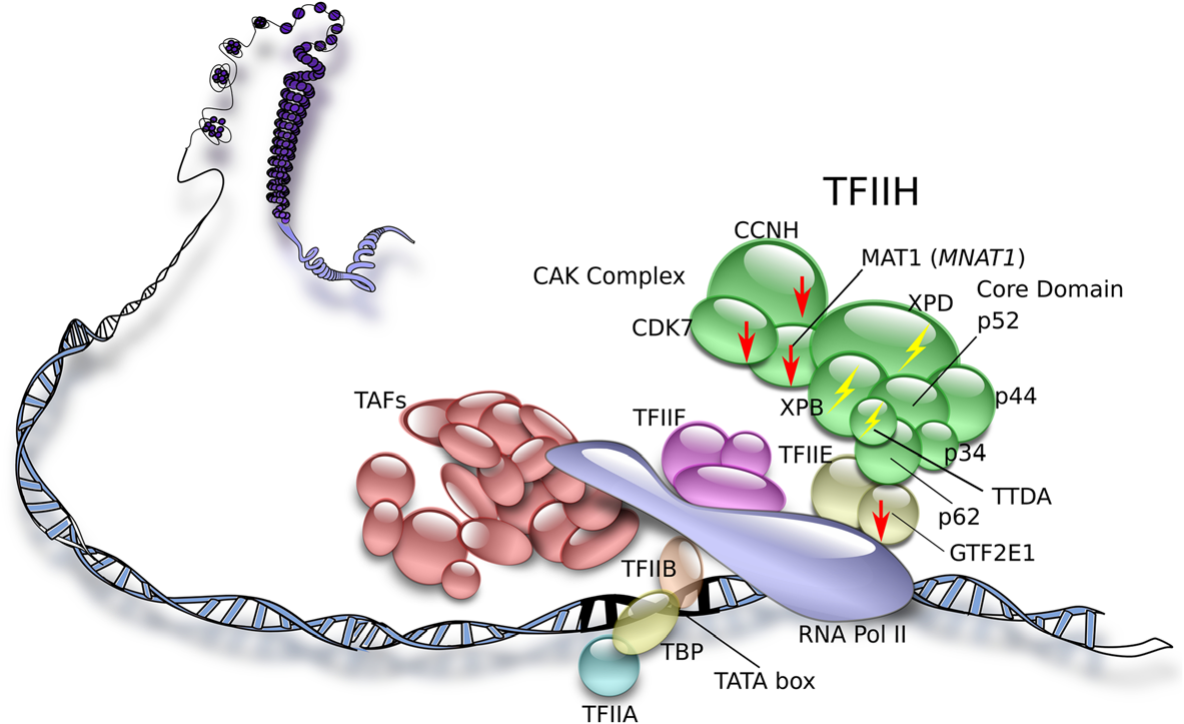
**Graphical depiction of our proposed mechanism of impairment of TFIIH-mediated function in transcription in placenta leading to preeclampsia.** We propose that impairment of TFIIH-mediated function in transcription, which may occur via several mechanisms such as mutations in the genes coding for subunits of its core domain (such as TTD-associated mutations in *XPD*, *XPB* and *TTD-A*), downregulation of the subunits of its CAK domain, and/or dysregulation of subunits of other transcription factors with which it interacts such as GTF2E1 of TFIIE, is one mechanism leading to preeclampsia. Red arrows depict downregulation. Yellow symbols depict mutations.

Our overall findings from our studies to date lead us to propose a mechanism, depicted in Figure 5, involving impairment of RNA Pol II-mediated transcription in placenta in association with gestational complications such as preeclampsia. We propose that impairment of TFIIH-mediated function in transcription, which may occur via several mechanisms such as mutations in the genes coding for the subunits of its core domain (such as TTD-associated mutations in *XPD*, *XPB* and *TTD-A*), downregulation of the subunits of its CAK domain, and/or dysregulation of subunits of other transcription factors with which it interacts such as GTF2E1 of TFIIE, is one mechanism leading to preeclampsia (Figure 5). Even though preeclampsia was not noted in pregnancies involving the three TTD patients with *TTD-A* mutations in our previous studies [8,36], we have included *TTD-A* in our proposed mechanism, given the risk of other gestational complications (such as decreased fetal movement) which may be associated with mutations in this gene.

Our proposed mechanism does not preclude other mechanisms which may be involved in preeclampsia development and may not explain all cases of preeclampsia. Taking advantage of the rich resource of publicly-available gene expression datasets, we designed a systems biology approach to select specific and relevant datasets for focused analyses addressing our study hypothesis. A limitation of this approach is that the secondary data used in our study were created to test different hypotheses than the one conceived by us. Thus, some of the datasets may not have been ideal for our study. For example, even though we tried to restrict our analyses to datasets which had biopsied placental tissues believed to be predominantly of fetal-cell origin, we cannot rule out the presence of mixed maternal and fetal cell-derived histology in some datasets (such as GSE5999 [22]) causing differential trophoblast composition.

Critical questions about our proposed mechanism remain to be answered and include the stage of placental development affected by TFIIH and/or TFIIE impairment potentially leading to preeclampsia. The initial defect leading to preeclampsia is generally believed to be poor extravillous trophoblast formation and/or inadequate remodeling of the maternal uterine spiral arteries, thereby compromising blood flow to the placenta causing placental ischemia-hypoxia and oxidative stress [3-7]. It is plausible that TFIIH and/or TFIIE impairment in placenta affect trophoblast proliferation, differentiation and/or migration creating the early defects which may lead to preeclampsia. A recent study examining transcriptional changes associated with differentiation from villous to extravillous first-trimester trophoblasts reported significantly higher expression of certain transcription-related factors such as JUN, ATF3, and EGFR in villous trophoblasts [39]. These findings hint at the relevance of these transcription factors to trophoblast differentiation and are consistent with our findings. ATF3 and EGFR were implicated as the main regulators of preeclampsia development in our previous integrative transcriptome analysis [19]. JUNB and JUND were highlighted in our current analyses as transcription regulators of the network involving the TTD genes, GTF2E1, *ATF3* and *EGFR*.

Another critical question which remains to be resolved pertains to the effect of hypoxia and oxidative stress on TFIIH and/or TFIIE function. In our previous study [19], *XPD* (*ERCC2*) was significantly down-regulated (FDR < 0.01, Fold Change = 1.65) in hypoxic human trophoblasts (Swan 71 human trophoblast cells under chemical hypoxia by CoCl2-treatment) in an *in vitro* system (GSE31679) [40]. In mouse studies, TTD/XPA double mutant cells were found to be hypersensitive to oxidative stress [41]. Recent findings of XPD’s involvement in apoptosis [42], make it theoretically plausible that TFIIH impairment could contribute to increased syncytialtrophoblast apoptosis/necrosis caused as the result of prolonged hypoxia and reoxygenation. The interrelationship of hypoxia and oxidative stress with TFIIH and/or TFIIE function remains to be explored in future focused experiments.

## Conclusion

Our investigations suggest an important role for TTD NER/transcription genes in placenta during gestational periods critical with respect to preeclampsia development and implicate impaired TFIIH-mediated function in transcription in placenta as a likely mechanism leading to preeclampsia. Our findings provide etiologic and mechanistic clues which can eventually be translated into therapeutic and/or preventive measures against preeclampsia.

## Methods

Raw data from relevant datasets were downloaded from GEO (http://www.ncbi.nlm.nih.gov/gds/). These included datasets containing global gene expression patterns of normal human tissue including placenta (GSE96) [21], time-course placenta from first, second and third trimester pregnancies (GSE5999) [22], and normal and preeclamptic placenta from a case-control study of preeclampsia (GSE10588) [23]. Other relevant datasets downloaded and analyzed included a human umbilical vein endothelial cells (HUVEC) transcriptome dataset (GSE27871) [27] created using siRNA-mediated knockdown of 400 factors, the reactome dataset [28], and raw data from the Encyclopedia of DNA Elements (ENCODE) [29] dataset.

### Analysis of NER/transcription gene profiles in normal human placenta

We conducted comparison of expression of NER/transcription genes in placenta versus other normal tissue including skin in GSE96 [21] via ANOVA models (Method of Moments [43]) using Partek software version 6.6 (Partek Inc., St. Louis, MO, USA). GSE96 contains global gene expression patterns (normal transcriptome) of 79 diverse human tissues including placenta. Placental biopsies were collected at term from four Caucasians ages 21-39 [21]. Temporal analysis of NER/transcription genes in placenta in GSE5999 [22] was done through Pearson correlation analysis, using time as a variable. GSE5999 contains global gene expression patterns of basal plate (maternal-fetal interface) of 36 placentas from 14 to 40 weeks gestation obtained at the conclusion of normal pregnancies.

### Placental gene expression profiles in a preeclampsia case-control study

The objective for analysis of GSE10588 [23] was to compare global gene expression profiles in placenta from normotensive pregnancies (controls) to placentas from preeclamptic pregnancies (cases). This dataset was the largest preeclampsia case-control study identified through GEO (http://www.ncbi.nlm.nih.gov/gds/) and included patterns of gene expression on tissue biopsies from fetal cell-derived histological subsections of the placenta as per our selection criteria. This dataset ascertained cases of severe preeclampsia as defined by the American College of Obstetricians and Gynecologists [44] occurring early-onset (i.e., diagnosis <34 weeks gestation) and matched case (n = 21) and control (n = 21) placentas based on gestational age.

GSE10588 was previously analyzed with respect to global differential gene expression patterns. We conducted a novel analysis on this dataset by using the Algorithm for the Reconstruction of Accurate Cellular Networks (ARACNe) [25] integrated into GeWorkbench (http://wiki.c2b2.columbia.edu/workbench) in order to infer transcriptional relationships and identify key gene regulators. ARACNE is an information-theory based method developed to reconstruct gene regulatory networks in mammalian cellular context using gene expression profiles [25]. ARACNE calculates Mutual Information (MI) between every combination of genes. If interactions (edges) between more than two genes (nodes) are found, ARACNE uses the Data Processing Inequality (DPI) algorithm to remove the weakest interaction. In our analyses, the threshold for MI was set at p = 10^-7^ and DPI was set at 0.15.

Further analysis of GSE10588 involved identifying protein-protein interaction sub-networks of the significant (q < 0.01, Fold Change ≥ 1.3) preeclampsia-specific gene list through application of Cytoscape [45] MiMI plugin [46] using the reactome dataset [28], employing direct-only protein-protein interactions as a filter. The reactome dataset [28] contains information on 2975 human proteins and 2907 protein-protein interactions.

### Transcriptome analysis of human umbilical vein endothelial cells

In order to compare the affected gene regulatory networks of GTF2E1 and FLT1, we analyzed raw data from a HUVEC transcriptome dataset (GSE27871) [27] created using siRNA-mediated knockdown of 400 specific signaling molecules and transcription factors. Correlation matrix for the 400 gene knockdown microarrays was created using Pearson correlation between individual knockdown datasets; an FDR cutoff of less than 0.05 was used. Correlation coefficient (R) was used as an input in the network construction and Edge-Weighted Spring Embedded layout algorithm was used to create the network. Gene clusters were identified via ClusterOne Cytoscape plugin [47] using default settings.

### Analysis of relevant datasets to identify common regulatory networks of genes implicated in preeclampsia

The reactome dataset [28] was also used for the identification of proteins and protein complexes with first- and second-degree interactions with GTF2E1.

In order to infer potential common transcriptional regulatory networks of selected genes and gene regulators implicated in preeclampsia, analysis of the ENCODE Chip Seq derived regulatory network data [29] was conducted in Cytoscape [48]. ENCODE contains genomic binding information of 119 transcription-related factors in >450 distinct experiments providing regulatory information crucial for understanding basic principles of human biology and disease.

## Endnote

^a^References for most *XPD* mutations analyzed in our previous report can be found in Table four of Cleaver et al. [49].

## Competing interests

The authors declare that they have no competing interests.

## Authors’ contributions

RM and AD conceived the study and analyzed the data. XA and VN helped with data analysis and interpretation. AK helped with data preparation and analysis. All authors read and approved the final manuscript.

## Supplementary Material

Additional file 1: Figure S1 NER/transcription gene expression profiles in normal human placenta. Panel a. Bar graph depicting expression (relative fluorescence intensity shown on log_2_ scale) of three TTD NER/transcription genes in normal human tissue array including placenta in GSE 96 [21]. Red bar represents the placenta and green bar represents the skin. Panel b. Temporal expression patterns of the three TTD NER/transcription genes in placenta from 14 to 40 weeks gestation in GSE5999 [22]. Circles represent individual placental samples. Click here for file
