# Supplementary figures and images for: Nucleotide excision repair/transcription gene defects in the fetus and impaired TFIIH-mediated function in transcription in placenta leading to preeclampsia
Source: BMC Genomics. 2014 May 15;15:373. doi: 10.1186/1471-2164-15-373 (PMC4229886; doi:10.1186/1471-2164-15-373)

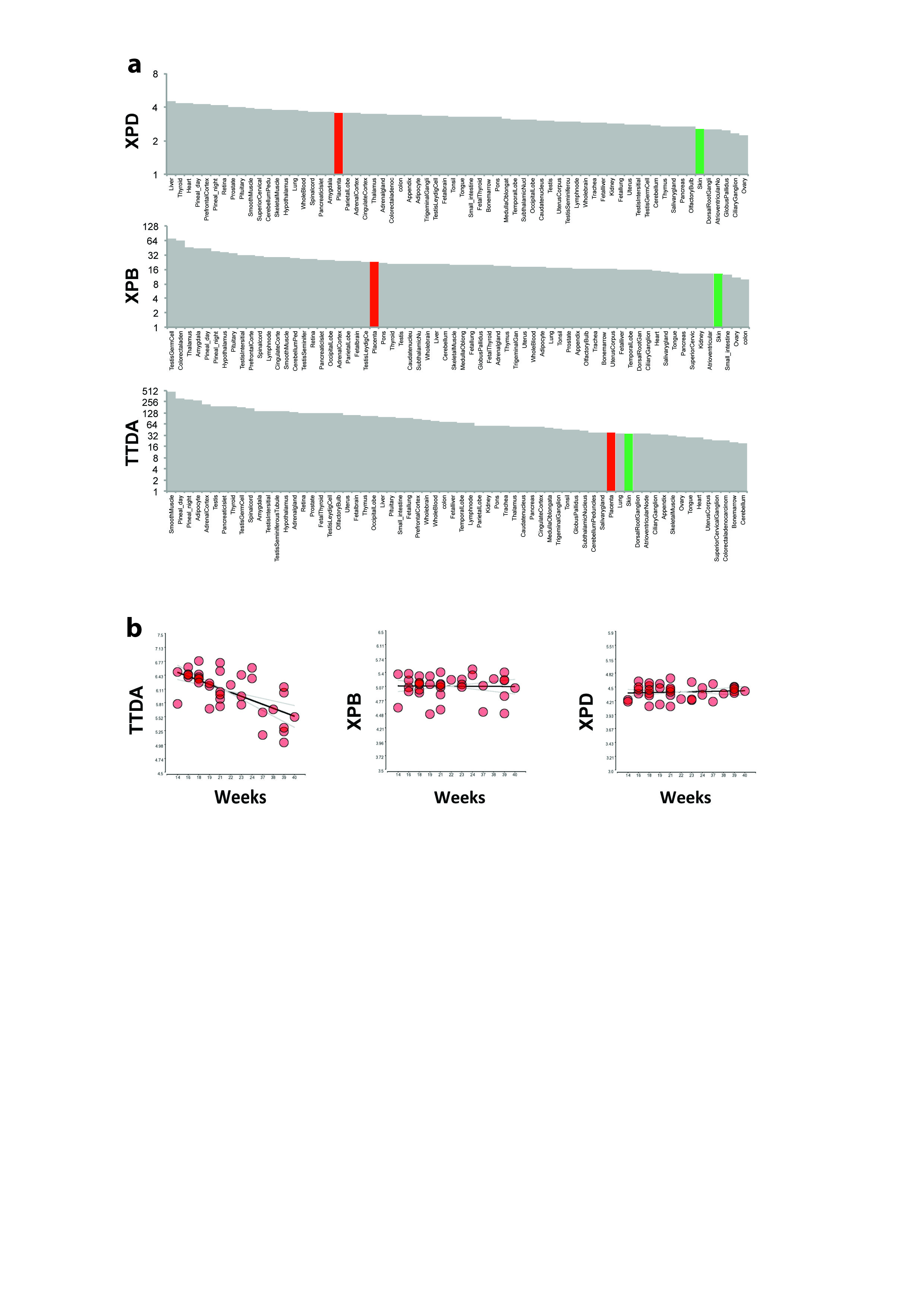

Supplement: Additional file 1: Figure S1 — NER/transcription gene expression profiles in normal human placenta. Panel a. Bar graph depicting expression (relative fluorescence intensity shown on log2 scale) of three TTD NER/transcription genes in normal human tissue array including placenta in GSE 96 [21]. Red bar represents the placenta and green bar represents the skin. Panel b. Temporal expression patterns of the three TTD NER/transcription genes in placenta from 14 to 40 weeks gestation in GSE5999 [22]. Circles represent individual placental samples. [file 1471-2164-15-373-S1.jpeg]
